# Patterns of Song across Natural and Anthropogenic Soundscapes Suggest That White-Crowned Sparrows Minimize Acoustic Masking and Maximize Signal Content

**DOI:** 10.1371/journal.pone.0154456

**Published:** 2016-04-29

**Authors:** Elizabeth P. Derryberry, Raymond M. Danner, Julie E. Danner, Graham E. Derryberry, Jennifer N. Phillips, Sara E. Lipshutz, Katherine Gentry, David A. Luther

**Affiliations:** 1 Department of Ecology and Evolutionary Biology, Tulane University, New Orleans, Louisiana, United States of America; 2 Biology Department, George Mason University, Fairfax, Virginia, United States of America; 3 Museum of Natural Science, Louisiana State University, Baton Rouge, Louisiana, United States of America; 4 Smithsonian Migratory Bird Center, Smithsonian Institution, National Zoo, Washington, DC, United States of America; Virginia Commonwealth University, UNITED STATES

## Abstract

Soundscapes pose both evolutionarily recent and long-standing sources of selection on acoustic communication. We currently know more about the impact of evolutionarily recent human-generated noise on communication than we do about how natural sounds such as pounding surf have shaped communication signals over evolutionary time. Based on signal detection theory, we hypothesized that acoustic phenotypes will vary with both anthropogenic and natural background noise levels and that similar mechanisms of cultural evolution and/or behavioral flexibility may underlie this variation. We studied song characteristics of white-crowned sparrows (*Zonotrichia leucophrys nuttalli*) across a noise gradient that includes both anthropogenic and natural sources of noise in San Francisco and Marin counties, California, USA. Both anthropogenic and natural soundscapes contain high amplitude low frequency noise (traffic or surf, respectively), so we predicted that birds would produce songs with higher minimum frequencies in areas with higher amplitude background noise to avoid auditory masking. We also anticipated that song minimum frequencies would be higher than the projected lower frequency limit of hearing based on site-specific masking profiles. Background noise was a strong predictor of song minimum frequency, both within a local noise gradient of three urban sites with the same song dialect and cultural evolutionary history, and across the regional noise gradient, which encompasses 11 urban and rural sites, several dialects, and several anthropogenic and natural sources of noise. Among rural sites alone, background noise tended to predict song minimum frequency, indicating that urban sites were not solely responsible for driving the regional pattern. These findings support the hypothesis that songs vary with local and regional soundscapes regardless of the source of noise. Song minimum frequency from five core study sites was also higher than the lower frequency limit of hearing at each site, further supporting the hypothesis that songs vary to transmit through noise in local soundscapes. Minimum frequencies leveled off at noisier sites, suggesting that minimum frequencies are constrained to an upper limit, possibly to retain the information content of wider bandwidths. We found evidence that site noise was a better predictor of song minimum frequency than territory noise in both anthropogenic and natural soundscapes, suggesting that cultural evolution rather than immediate behavioral flexibility is responsible for local song variation. Taken together, these results indicate that soundscapes shape song phenotype across both evolutionarily recent and long-standing soundscapes.

## Introduction

The soundscape includes all sounds in a landscape [1] and is the backdrop against which all acoustic communication takes place. This backdrop can pose limitations when noise masks (i.e., limits perception of) signals used in communication [2]. Selection thus favors the ability of an organism to detect and discriminate signals in noise [3–6]. Recent studies demonstrate that anthropogenic noise pollution affects both vertebrate [7–12] and invertebrate [13] acoustic communication, with most evidence from studies on avian communication [14]. Many species of birds produce songs with higher minimum frequencies in urban areas compared to rural areas, presumably to be heard over the high amplitude, low frequency noise generated by machines [11, 12, 15–21]. Further, birds with relatively low frequency vocalizations appear to avoid areas with anthropogenic noise in the same bandwidth as their songs [22, 23], suggesting that soundscapes can act as species filters. In contrast, we know relatively less about variation in acoustic signals in natural soundscapes [6].

Like human-generated noise, natural sounds can be high amplitude at low frequencies and potentially mask communication signals. Natural abiotic high amplitude, low frequency sounds include running water, surf, rain, wind and wind-blown vegetation. The limited evidence available in birds in the context of high levels of natural ambient noise suggests higher calling rates in wind [24], serial redundancy near waterfalls [25], or an overall reduction in singing behavior in rain [26]. Unlike the findings in anthropogenic soundscapes, there is no strong evidence that birds sing higher frequency songs in higher levels of low frequency natural abiotic noise. A systematic approach is therefore needed to understand how organisms are responding to both evolutionarily long-standing (natural) as well as recently altered (anthropogenic) soundscapes [27].

Vocalizations are often described based on their 'communication distance,' i.e., the distance at which other organisms can detect or discriminate the signal from background noise [28]. Bird song is a long-distance communication signal [29], and, as such, a decrease in communication distance can affect a song’s function in the contexts of both mate choice and male-male competition. Communication distance depends on the auditory processing abilities of an organism, the type of signal produced, and the sound environment between signaler and receiver [30]. Thus, in considering behavioral responses to the sound environment, we need to assess not only how songs vary within and across soundscapes, but also how background noise levels affect the audible frequency range for receivers.

Changes in song behavior to avoid masking from background noise could occur through a number of mechanisms, including 1) cultural evolution and 2) immediate flexibility. A genetically evolved response of song phenotype to noise is another possible mechanism [27], but for birds that learn their song, more work is needed to understand the genetic template of song before testing this mechanism. During cultural transmission of song, noise may influence which songs, or parts of songs, that young birds learn [14]. In noisy environments, for instance, birds may only learn to sing higher frequency notes because they cannot detect lower frequencies in the presence of the high amplitude, low frequency noise characteristic of cities and running water [14]. Alternatively, males may be able to detect lower frequencies but selectively copy higher frequency songs because they are less degraded by noise, rather than lower frequency songs that are degraded [31–33]. Thus, songs would evolve over generations (via cultural evolution) in response to acoustic selection pressures [32]. In contrast, an immediate flexible response involves birds adjusting aspects of their songs, such as the frequency and/or amplitude, in real time in response to noise [34], which has now been demonstrated in a number of species [14, 15, 35–41]. For example, urban great tit (*Parus major*) males selectively sing song types with higher minimum frequencies within their repertoire in response to urban noise [38]. Cultural evolution and immediate flexibility are not alternative hypotheses, however, and both may explain variation in song and/or occur in different situations [14, 34]. Exploring the relative roles of cultural evolution and immediate flexibility, as well as the potential synergy between these two mechanisms is a critical component missing from the current understanding of how soundscapes affect acoustic communication systems.

In this study, we measured differences in song minimum frequency among individual male white-crowned sparrows (*Zonotrichia leucophrys*) holding territories in sites across a noise gradient in both urban and rural areas. First, we hypothesized that songs are adapted to local soundscapes, and predicted that song minimum frequencies would i) be higher at locations with higher amplitude noise, and ii) be equal to or higher than the predicted lower frequency limit to hearing at a given site when accounting for both physical limits to hearing for a species and masking from background noise. Second, we hypothesized that song differences resulted from cultural evolution rather than immediate flexibility because white-crowned sparrow males produce only one, highly stereotyped song type [42], which may limit capacity for immediate flexibility. We therefore predicted that the average ambient noise level in a region ('site noise') would better predict song minimum frequency than the noise level on an individual's territory. This prediction rests on the assumption that cultural evolution will be detectable at the site level because natal dispersal following song learning would have a homogenizing effect on the spatial distribution of song frequencies within sites, whereas immediate flexibility will be detectable at the territory level because of spatial variation in noise within sites.

We conducted this study at two spatial scales, including four separate analyses. First, at an urban local scale, we tested our hypotheses within a cultural population consisting of one city park with three sites that differ in noise levels, where all birds sang the same song dialect and where that dialect has been found for at least thirty years [43]. These sites share similar histories of changes in song over time (see [43]) and therefore provide the most controlled test of song differences in relation to noise. Second, at a regional scale, we included urban and rural sites and asked if noise affects song similarly across anthropogenic and natural soundscapes. In addition, the study sites encompassed multiple dialects (i.e., cultural populations), which provide replication in our tests. Third, we tested our hypotheses among rural sites alone to ensure that regional patterns were not driven solely by an urban vs. rural dichotomy of noise and song phenotypes. Last, we asked if noise masks song minimum frequencies by comparing song frequencies to modeled masking profiles at one rural and four urban sites for which we had data sufficient to generate these profiles.

## Methods

We studied Nuttall’s white-crowned sparrows (*Zonotrichia leucophrys nuttalli*; NWCS) as a model organism for song evolution. NWCS is a year-round resident in both urban and rural areas in and around San Francisco, CA, and is known to have small dialectal regions that result from cultural evolution [44–46]. Retrospective studies of urban sites provide evidence that NWCS songs have adapted culturally to increases in noise from traffic over time [43, 47].

**Fig 1:**
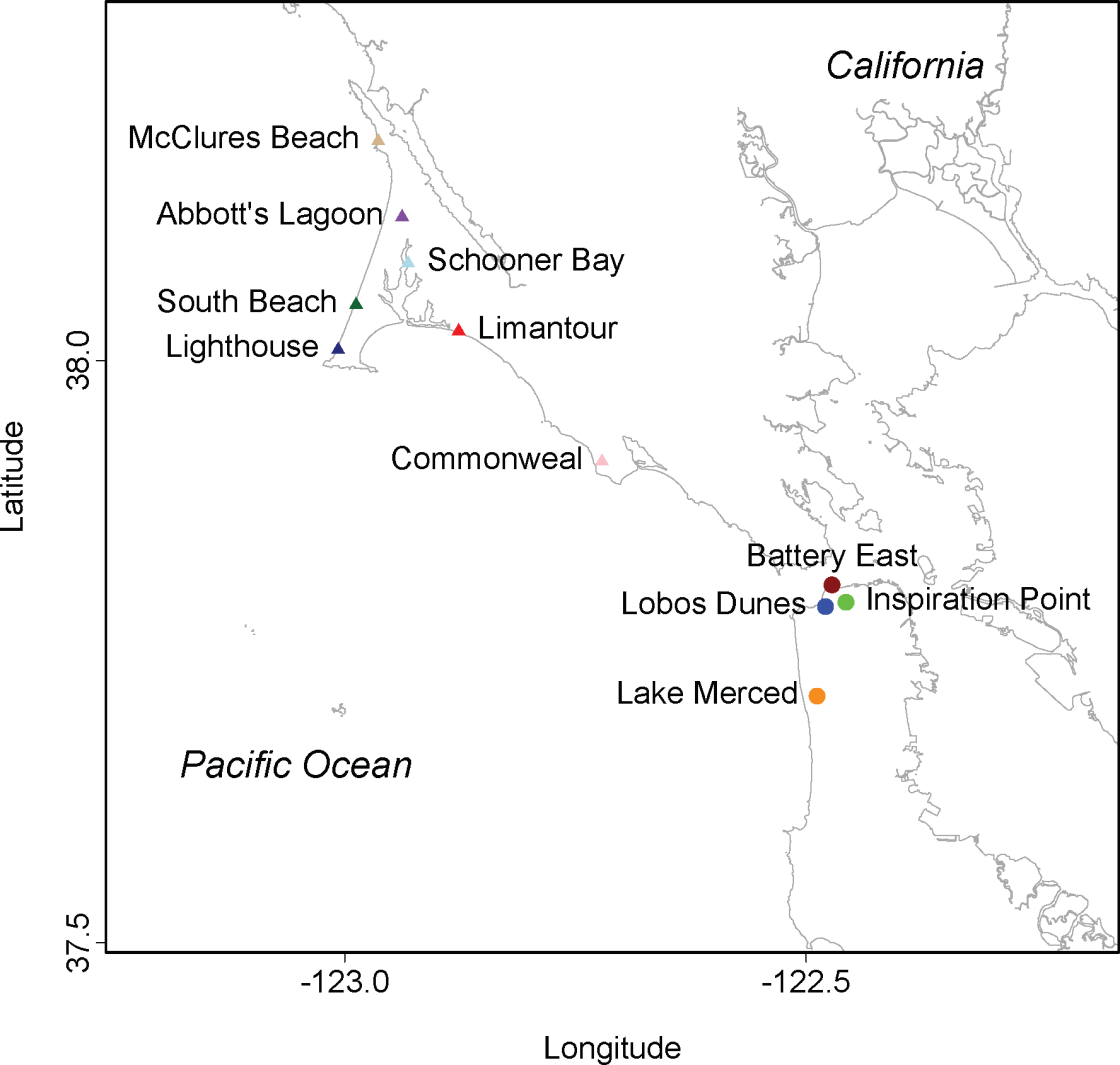
Map of study sites. Circles indicate sites within city limits of San Francisco, CA, U.S.A. Triangles indicate sites outside city limits. See text for details on each site.

We recorded the songs of territorial male NWCS and the background noise levels on their territories at a set of urban and rural sites in San Francisco and Marin counties in California (Fig 1). Sites were designated as ‘urban’ if they were within San Francisco’s city limits, whereas sites outside the city limits were designated as ‘rural’. Urban sites included Lobos Dunes (n = 16 males), Inspiration Point (n = 14), Battery East (n = 11), and Lake Merced (n = 15); rural sites included Commonweal (n = 8), Abbott’s Lagoon (n = 9), Lighthouse (n = 4), Limantour (n = 6), McClures Beach (n = 4), Schooner Bay (n = 7), and South Beach (n = 4). Hereafter, we refer to sites with the notation U to assign the urban sites and R to assign the rural sites followed by the site name in subscript. All recordings were made between 9 July 2013–16 July 2013 and 5 May 2014–16 May 2014 during the breeding seasons.

### Background noise recording and measurement

We measured background noise levels at each territory following the methods developed by Brumm [48]. We recorded two minutes of background noise using a Sennheiser ME62 omnidirectional microphone mounted facing upwards 1m above the ground on a tripod and a Marantz PMD661 digital recorder set at the same gain across all recordings. To calibrate the sound pressure levels in each noise recording, we simultaneously measured the maximum sound pressure twice at each of the four compass points for 10s, for a total of eight values per territory. We measured sound pressure levels using a 407736 Extech Sound Level Meter (response time = 125ms, accuracy = ± 1.5dB, weighting = A) mounted 1.5 m above the ground on a tripod, which is a typical perching height for NWCS in this habitat (pers. obs.).

To characterize the 'average' value of the background noise for each territory, we calculated the equivalent continuous noise level from the eight sound pressure values (LAeq) [49]. Because we were interested in the sustained noise that birds experience rather than brief loud noises, we dropped sound pressure levels caused by loud, intermittent noise sources, such as a plane flying overhead. To estimate the average noise per site, we averaged all LAeq values per site.

### Predicted minimum song frequencies based on hearing and masking profiles

We first estimated an audibility curve (i.e., an audiogram), which is the minimum sound amplitude that an animal perceives across its range of hearing. The audibility curve was based on previously published measurements of auditory brainstem responses of nine individual *Z*. *leucophrys* in response to a range of sound frequencies (500–7,000Hz) in a quiet setting [50, 51]. We modeled the audibility curve with a nonlinear mixed-effects model fit by maximum likelihood using function nlme [52] in R [53] as follows:

$$Hearing\;threshold=Minimum\;amplitude\;of\;hearing-\alpha +\alpha \;\times \;\sqrt{1+{\left(\text{log}(Frequency)-\;\text{log}(Peak\;Hearing\;Frequency)\right)}^{2}}\;,$$

where *α* is a fitting parameter for the hyperbolic model, *Minimum amplitude of hearing* was the lowest amplitude (in dB) that elicited an auditory brainstem response at a given *Frequency* for each individual bird and was treated as a random effect. *Peak Hearing Frequency* was the frequency of most sensitive hearing (i.e., the frequency of the lowest amplitude that elicited a response).

We then created noise profiles for each of five core study sites (U_Lobos Dunes_, U_Inspiration Point_, U_Battery East_, U_Lake Merced_, and R_Commonweal_). The core sites consist of the urban sites and one rural site, and have been the focus of research for many years [43, 44, 47, 54]. We examined all four urban sites and one rural site closest in geographic proximity. For each site, we calibrated and estimated a noise spectrum from the background noise recordings described above. We calibrated the noise spectra with the paired sound pressure levels using the Sound Level Meter function in SIGNAL 5 software [55] and dropping outliers. We averaged sound pressure levels across 1/3 octave bands in 16 adjacent bands between 1,000 and 10,000 Hz. To identify the critical threshold (i.e., the amplitude of noise that would mask other sounds) at each frequency band, we applied a masking function (i.e., critical ratio). A masking function describes the sound pressure level in decibels that is necessary for a sound to be heard over a background noise. Because masking functions have not been calculated for white-crowned sparrows, we used the masking function that was measured empirically for a closely related species, the song sparrow (*Melospiza melodia*) [56]. We interpolated critical threshold values with a spline function. Last, we generated a masking profile for each of the five sites by taking the higher amplitude of the audibility curve or the critical threshold for the range of frequencies.

### Song recording and measurement

We recorded between 1 and 25 songs (average 7.76) from each territorial male in a single recording session. Males sing approximately 6 songs per minute, and most recording sessions lasted less than 8 minutes. Because NWCS songs are highly stereotypic, measurements from one song per individual are sufficient to describe song features [57]; however, if more than one song was recorded for an individual male in a session, we averaged measurements across recordings. Recordings were made within 8–16 meters of the focal bird using a Sennheiser ME62 omnidirectional microphone mounted on a Saul Mineroff PR-1000 parabola and a Marantz PMD 661 MKII digital recorder set at 44.1 kHz sampling rate, 16-bit, and WAV file type. We used SIGNAL 5.0 for sound analysis. Prior to analysis, we resampled songs at 25 kHz. We measured minimum frequency from spectrograms (256 point fast Fourier transform, frequency resolution = 98 Hz, time resolution = 10.2 ms) at -36 dB relative to the peak amplitude frequency in the song (Hann transformation, transform length = 256, console window height = 0.1). Because frequency measures were taken at a fixed number of decibels below the peak amplitude, variation in frequency measures was not due to variation in song amplitude [58].

### Statistical Analyses

To test if song minimum frequencies were related to background noise level, and to assess support for the cultural selection hypothesis versus the immediate flexibility hypothesis in causing this pattern, we used an information theoretic approach following Burnham et al. [59]. We conducted the analyses at two spatial scales. On a local scale, we studied three urban sites that are located within one dialect zone and within one city park. On a regional scale, we conducted one analysis with all urban and rural sites and another analysis with only rural sites. We built several linear models of song frequency as a function of background noise level and selected the best descriptive models based on how well they fit the data using AIC_c_ (Akaike's Information Criterion [60]). The background noise level variable differed among models. To test if songs vary with local site noise level, we included site noise level in some models. To test if songs vary more locally in response to territory noise level, we included territory noise level in some models. Based on plots of raw data, which suggested that minimum frequencies level off at the loudest sites, we included quadratic terms of either site or territory noise level in some models. Preliminary analyses indicated that year had no effect on measurements, so year was not included in further analyses. In separate analyses, we further tested if songs are adjusted to territory noise level at each site by testing the fit of models with additive and interactive effects of site and territory noise level. All raw data are provided in 'S1 File', and all scripts for analyses can be found in 'S2 File'.

We present effect sizes (B) ± standard errors, correlation coefficients (R^2^), and measures of support for models, including the weight of the model of interest (w_i_) and the evidence ratio (ER = w_i_/w_null model_), which is interpreted as the probability that the model of interest is the best model in the set as compared to an appropriate null model. We performed all analyses with R [53] and implemented the information-theoretic approach with package AICcmodavg [61].

To determine if site noise masks song minimum frequency, we plotted and visually compared mean minimum song frequencies and masking profiles for each site. First, we plotted the audibility curve (i.e., hearing threshold in silence) following [62] for the *Z*. *leucophrys* individual with the best measured hearing in [50]. We chose to use the audibility curve of the *Z*. *leucophrys* with the best hearing, because it predicts the lowest minimum frequency a bird may be capable of hearing at each site. We also plotted the masking profiles for each site. We then plotted a line 12dB above the best (i.e., lowest) intensity of the audibility curve. Where the masking profile of each site crossed this line is the predicted low frequency limit of hearing at that site. Lastly, we plotted the observed mean song minimum frequency of each site to compare against these predicted values. Observed values higher in minimum frequency than the predicted values would support our hypothesis that songs of NWCS vary spatially to transmit through the local soundscape.

### Ethics Statement

The field studies did not involve endangered or protected species. Tulane Institutional Animal Care and Use Committee and National Park Service Institutional Animal Care and Use Committee approved this research. The U.S. Fish and Wildlife Service (MB679782-1), the Bird Banding Laboratory (23900), and the California National Resources Agency (SC-6799) granted permission for this research. Permission for research in the urban sites was granted by the Golden Gate National Recreation Area (SCI-0007), the San Francisco Parks and Recreation (041415), and The Presidio Trust and in the rural sites by the Point Reyes National Seashore (SCI-0016).

## Results

**Fig 2:**
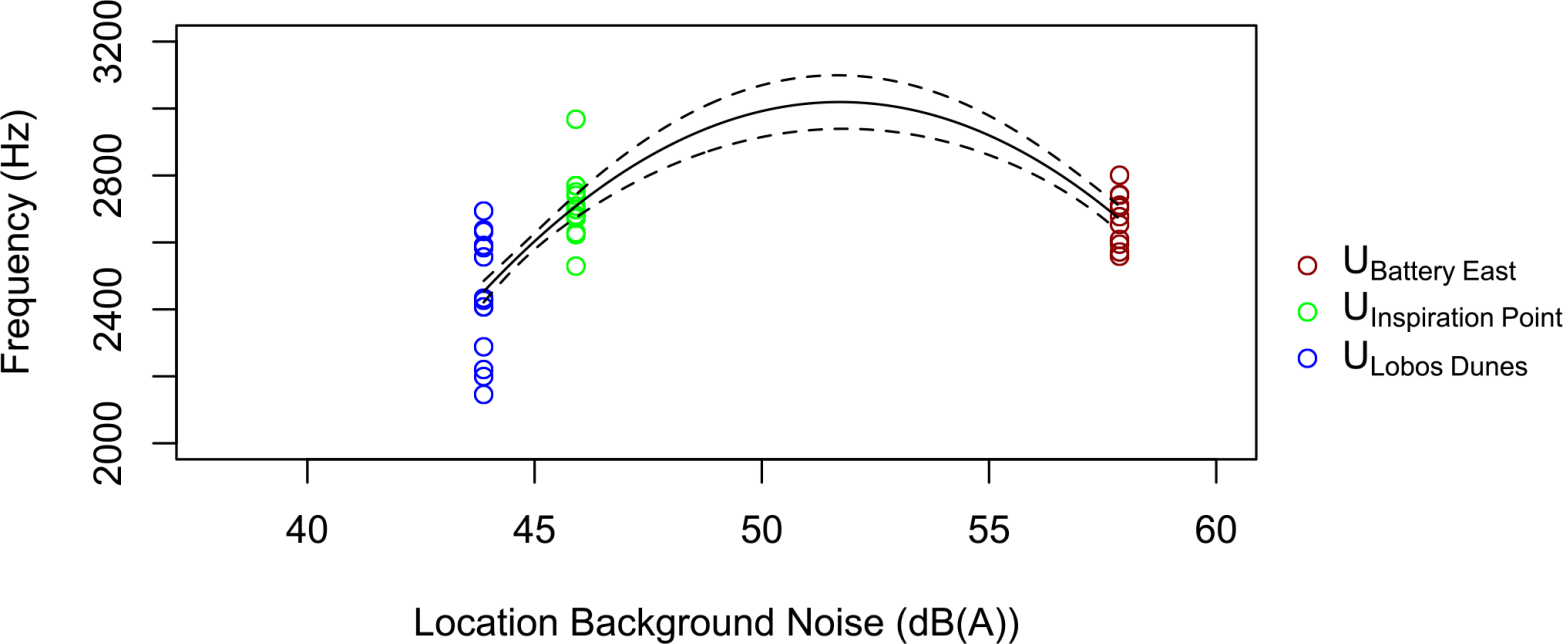
Song minimum frequency is related to background noise levels in the same urban dialect. Song minimum frequency is on the y-axis and the amplitude of background noise is on the x-axis. The San Francisco dialect was sampled at three urban locations, U_Battery East_, U_Inspiration Point_, and U_Lobos Dunes_. The solid line represents model average predictions and the dashed line represents the standard error.

**Fig 3:**
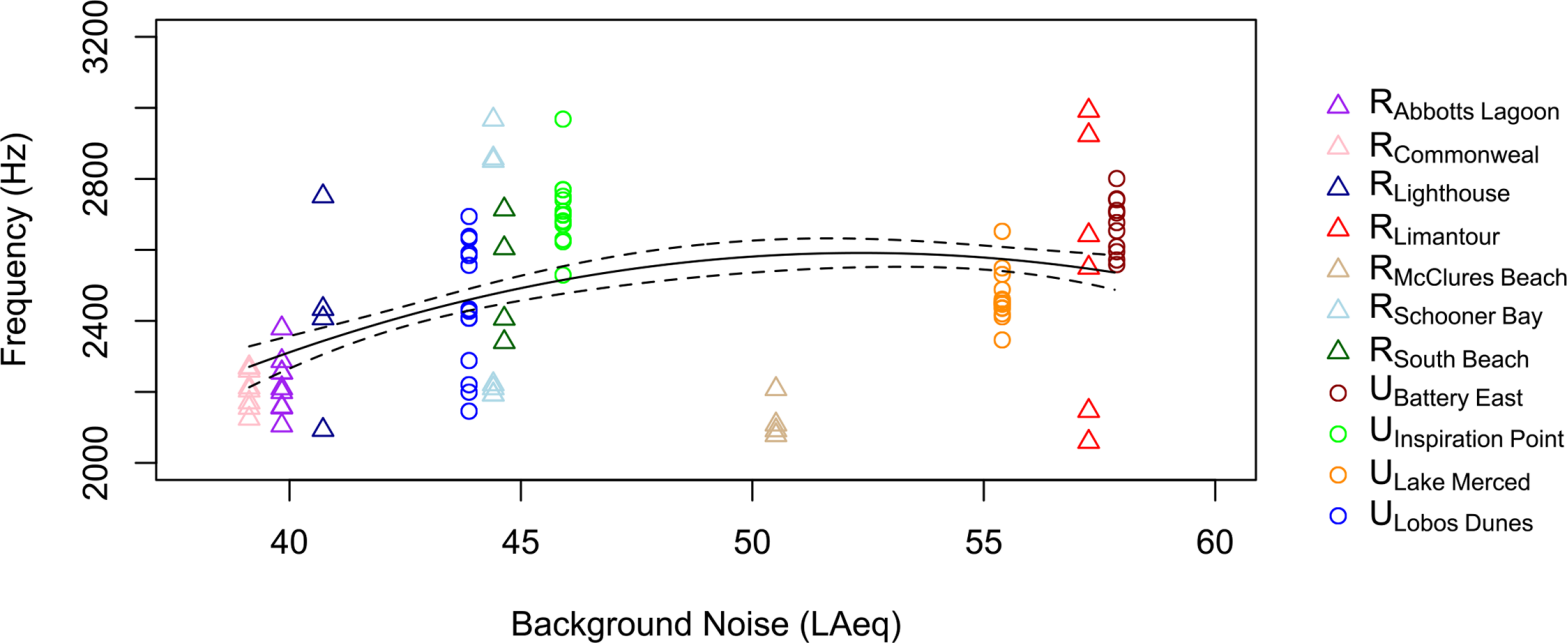
Song minimum frequency is related to background noise levels across all sites. Song minimum frequency is on the y-axis and the amplitude of background noise is on the x-axis. Urban locations included the San Francisco dialect sampled at U_Battery East_, U_Inspiration Point_, and U_Lobos Dunes_ and the U_Lake Merced_ dialect sampled at Lake Merced. See text for dialects at rural locations including R_Commonweal_, R_Abbott's Lagoon_, R_Lighthouse_, R_Schooner Bay_, R_South Beach_, R_McClures Beach_, and R_Limantour_. The solid line represents model average predictions and the dashed line represents the standard error.

**Fig 4:**
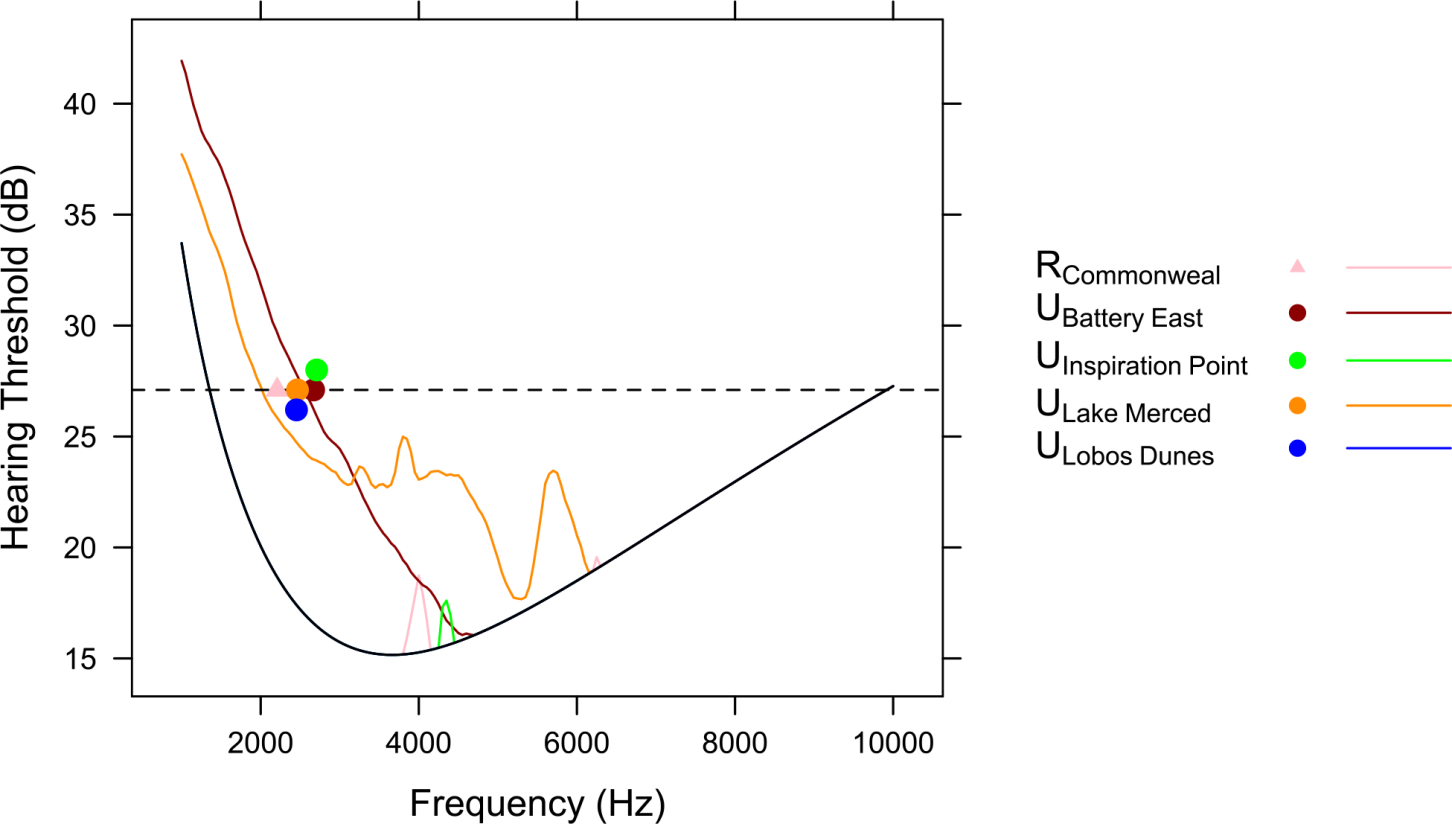
Sound masking profiles and song minimum frequencies for five sites. The solid black line represents the audibility curve (i.e., hearing threshold in silence; following Dooling 2007) for *Z*. *leucophrys*. Colored lines are masking profiles for each site, and are shown only where they occur above the audibility curve. The dashed line represents 12dB above the best (i.e., lowest) intensity of the audiogram of the bird with the best hearing. Where the masking profile crosses the dashed line is the predicted low frequency limit of hearing. The mean song minimum frequency for each site is plotted on the dashed line. Points for two sites are shifted up or down from that line for visual clarity. At each site, the minimum song frequency is higher than the predicted low frequency limit of hearing, and this is most distinctive for two sites: U_Battery East_ and U_Lake Merced_ whose masking profiles cross the dashed line at higher frequencies than the audibility curve. The other three sites have predicted frequency limits much lower than the observed song minimum frequencies (U_Lobos Dunes_, U_Inspiration Point_, and R_Commonweal_).

Background noise levels varied widely across the study sites (w_i_ of model with site = 99.99%, R^2^ = 0.69; Figs 2 and 3). Urban sites typically had higher background noise levels than rural sites, particularly at low frequencies (Fig 4). Background noise levels for both urban and rural sites corresponded to noise profiles measured by the National Park Service (NPS) Natural Sounds Program which monitored background noise level at two sites near our urban and rural areas for 30 days each (55.5 dBA and 44.9 dBA respectively [63, 64]). The Natural Sounds Program found that the urban soundscape was dominated by noises from vehicles, fog horns from the Golden Gate Bridge, people, and dogs, in addition to wind [64] whereas the rural soundscape was dominated by noises from wind, wind blowing through grass, and surf [63]. We also found that noise sources differed between urban and rural sites. In urban areas, noise resulted mainly from traffic, whereas rural sites experienced noise from both human activity and natural sources.

All urban males recorded in U_Battery East_, U_Inspiration Point_, and U_Lobos Dunes_ produced song types that fall within the typical variation of the 'San Francisco' dialect as described by Baptista [43, 44]. Males at U_Lake Merced_ produced songs of either the 'San Francisco' dialect or a combination of the 'San Francisco’ and ‘Lake Merced' dialects. Four males in U_Lobos Dunes_ inserted a new complex note (a stuttered buzz) between the initial whistle and main buzz. The new note did not influence song minimum frequency, and the remainder of the song was typical of the SF dialect. Males recorded in Marin County (all rural sites) generally produced song types consistent with dialects described by Baker and Thompson [65]. Males in R_Commonweal_ sang the 'Clear' dialect, in R_Abbott's Lagoon_ sang mixed 'McClure' and 'Drake' dialects, R_Lighthouse_ sang 'Barries Bay' and 'McClure' dialect, R_Limantour_ sang 'Limantour' dialect, R_McClure_ sang 'McClure' dialect, R_Schooner Bay_ sang 'Drake' dialect, and R_South Beach_ sang 'Barries Bay' dialect.

Across the three urban sites that belong to the same cultural population, song minimum frequency was positively related to site noise level, and leveled off at noisier sites (Fig 2). The top ranked model included site noise level and site noise level^2^, and garnered 99.9% of the model weight, providing strong support for that model (Table 1). Evidence that minimum frequency positively correlated with site noise level was strong (ER > 48,000, B = 954 Hz / LAeq ± 186 s.e.). Models with territory noise level were ranked below those with site noise level (ERs < 1), providing evidence that song varies with site noise level rather than territory noise level. The top model (which included site noise level and site noise level^2^) also showed strong support for the quadratic term of site noise level (ER > 11,000, B = -9 Hz / LAeq ± 2). The top model explained 47% of the variation in the song data (R^2^ = 0.47). Plots of minimum frequency vs. noise level at individual sites showed no positive or negative relationships, and models with additive and interactive effects of site and territory noise level were not supported (ERs < 1).

**Table 1:** Rank of models that describe song minimum frequency across three urban sites, where all birds sing the same dialect.

Model	K	AICc	ΔAICc	w_i_
Site noise level + Site noise level^2^	4	520.3	0	0.999
Site noise level	3	539.0	18.7	1.0e^-4^
Intercept only (null) model	2	541.9	21.6	0
Territory noise level	3	542.4	22.1	0
Territory noise level + Territory noise level^2^	4	542.8	22.5	0

All candidate models are shown; K, number of parameters in model; AICc, Akaike information criterion with a correction for finite sample sizes; ΔAICc, difference between each model's AICc and that of the best model; w_i,_ model weight

Across all sites and dialects, the regional pattern matched what was found at the local urban sites from the same cultural population: song minimum frequency was positively related to site noise level, and minimum frequency leveled off at noisier sites (Fig 3). The top ranked model included site noise level and site noise level^2^, and showed strong support for site noise level, garnering 91% of the model weight (Table 2). Evidence that minimum frequency rose with background noise levels was strong (ER = 44, B = 205 Hz / LAeq ± 70). As found among the urban sites, models with territory noise level were ranked below those with site noise level (ERs < 1), providing evidence that song varies with site noise level rather than territory noise level. The top model (with site noise level and site noise level^2^) showed strong support for a quadratic term of site noise level (ER = 13, B = -2 Hz / LAeq ± = 0.7). The top model explained 18% of the variation in the song data (R^2^ = 0.18). Plots of minimum frequency vs. noise level at individual sites showed no positive or negative relationships, and models with additive and interactive effects of site and territory noise level were not supported (ERs < 1).

**Table 2:** Rank of models that describe song minimum frequency across all sites.

Model	K	AICc	ΔAICc	w_i_
Site noise level + Site noise level^2^	4	1339.2	0	0.91
Site noise level	3	1344.4	5.2	0.07
Territory noise level + Territory noise level^2^	4	1346.8	7.6	0.02
Territory noise level	3	1350.9	11.8	2.5e^-3^
Intercept only (null) model	2	1354.6	15.4	4.1e^-4^

All candidate models are shown; K, number of parameters in model; AICc, Akaike information criterion with a correction for finite sample sizes; ΔAICc, difference between each model's AICc and that of the best model; w_i,_ model weight

Across rural sites alone, song minimum frequency tended to be positively related to site noise level (Fig 3). The top ranked model included site noise level and garnered 43% of the model weight. Evidence that minimum frequency positively correlated with site noise level was weak (Table 3, ER = 2, B = 13 Hz / LAeq ± 7 s.e., R^2^ = 0.09). Models with territory noise level were ranked below those with site noise level (ERs < 1) and models with quadratic terms were not supported (ERs < 1).

**Table 3:** Rank of models that describe song minimum frequency across all sites.

Model	K	AICc	ΔAICc	w_i_
Site noise level	3	591.5	0	0.43
Intercept only (null) model	2	593.0	1.4	0.21
Territory noise level	3	593.6	2.1	0.15
Site noise level + Site noise level^2^	4	593.7	2.1	0.15
Territory noise level + Territory noise level^2^	2	595.5	4	0.06

All candidate models are shown; K, number of parameters in model; AICc, Akaike information criterion with a correction for finite sample sizes; ΔAICc, difference between each model's AICc and that of the best model; w_i,_ model weight

The noise level frequency ranges that are audible to NWCS varied among the five core sites (Fig 4). For four of these sites, the noise levels were enough to yield a masking profile that was greater than the audibility curve. In U_Battery East_ and U_Lake Merced_, the noise levels were sufficient to increase the lower frequency limit of hearing, and the noise sources were anthropogenic. In R_Commonweal_ and U_Inspiration Point_, the effect on hearing was at higher frequencies (circa 4kHz), and the noise source was most often songs of other birds. In all five sites, the mean song minimum frequencies were higher than the predicted lower limits of hearing.

## Discussion

Here we investigated the patterns and processes of song evolution in both natural and anthropogenic soundscapes for one species, white-crowned sparrows. We provided evidence that bird song varies with background noise levels across a range of both urban and rural soundscapes. Male white-crowned sparrows produced songs with higher minimum frequencies at sites with higher background noise levels, whether the noise source was natural or anthropogenic. In addition, song minimum frequency was produced within the optimal hearing range of intended receivers within sites. Together, these findings suggest that song phenotype varies with the soundscape to avoid masking by ambient noise. However, variation in song minimum frequency appeared constrained, as we found an upper limit on minimum frequency at both the local and regional scale. In considering the processes generating these patterns, our findings provide support for cultural selection over immediate flexibility as the potential mechanism driving the association between song minimum frequency and ambient noise level. Altogether our study provided the first empirical evidence within an organism of similar patterns and processes underlying response to masking by ambient noise in both natural and anthropogenic soundscapes.

Our analyses at both the local (within a cultural population) and regional (across cultural populations) scales find that male white-crowned sparrows produced songs with higher minimum frequencies at sites with higher background noise levels. Our evidence is strongest within a cultural population within an anthropogenic soundscape, probably because this sampling included some of our loudest sites, and these sites had the same cultural history. This finding is consistent with previous studies demonstrating that in a number of species, birds on territories with higher levels of human-generated noise produce songs at higher minimum frequencies (reviewed in [14]), although we find that site noise level is a better predictor of song minimum frequency than is territory noise level. We also find good evidence for higher song minimum frequencies at louder sites at a regional scale, supporting a similar pattern across cultural replicates and in both natural and anthropogenic soundscapes. Within natural soundscapes alone, we find a positive, albeit weak, relationship between song minimum frequency and noise level. The pattern may have been weak among rural sites alone because of the large number of dialects (i.e., different cultural trajectories) and smaller sample size compared to the analysis that included all sites. Although the pattern was weaker, our natural soundscapes provided some of the most extreme measures of ambient noise levels and song minimum frequency. For example, some rural sites, including McClures Beach and Limantour, included territories with particularly high amplitude, low frequency noise from ocean waves. Some of the birds from these rural sites had our highest recorded minimum frequency values, suggesting a response to ocean-generated noise. In comparing our findings to previous studies, there is mixed evidence to date of how birds respond to masking by natural abiotic sound sources. Although a *Phylloscopus* warbler species found near waterfalls tends to produce high-pitched vocalizations in comparison to other closely-related species [66], a study on chaffinches found that most males near waterfalls do not produce songs with significantly higher song minimum frequency [25]. Our findings suggest that song phenotype does vary with natural and anthropogenic sound sources, but that more work is needed to examine avian vocal strategies, particularly in natural soundscapes.

In our masking analysis, we found that observed song minimum frequency was higher than the predicted lower frequency limit of hearing and within the optimal hearing range of intended receivers. This finding provides additional support to our hypothesis that spectral variation in songs across soundscapes is to avoid masking by high amplitude, low frequency noise. We had five core sites with data sufficient to estimate masking profiles. Two urban sites had similar overall ambient noise levels (56 and 58 dB(A)) but different song minimum frequencies. This difference may be due to different shapes to their noise profiles. The difference in noise profiles resulted in different expected lower frequency limits to hearing in the two sites, such that noise in U_Battery East_ was predicted to mask song at higher frequencies than in U_Lake Merced_ (Fig 4). As predicted, males in U_Battery East_ produced songs at higher minimum frequencies than in U_Lake Merced_, and males in both locations produced songs on average at minimum frequencies above their respective hearing limits (Fig 4). These differences in song minimum frequency could improve transmission at their respective sites [67]. The other three core sites also had song minimum frequencies higher than the predicted lower limits of hearing, but in these sites, noise profiles did not greatly affect the lower limit of hearing. Noise levels were enough to yield a masking profile that was greater than the audibility curve, suggesting that noise sources at these sites do affect how NWCS songs are being heard by conspecifics. Although we were not able to generate masking profiles for all sites, such future analyses may help explain differences in song minimum frequency at sites with similar ambient noise levels.

Variation in song minimum frequency appeared constrained as, at the noisiest sites, we found that minimum frequencies leveled off near 2600 Hz. This result suggests that there is an upper limit to minimum frequencies in noisy soundscapes. If a limit to minimum frequencies exists, the source of limitation is most likely behavioral rather than mechanical given that this paper and others [65] have shown that male NWCS are physically capable of producing higher minimum frequencies. Behavioral constraints on song minimum frequency might be caused by sexual selection on frequency bandwidth [68, 69], as male NWCS respond more strongly to songs with wider bandwidth [54]. Males on territories with higher ambient noise levels tend to produce higher minimum frequencies as well as narrower bandwidths [54]. If increasing song minimum frequency leads to a reduction in bandwidth, then males may pay a cost in terms of territorial interactions. Thus, males may be balancing the benefits of increasing communication distance with the costs of signaling weaker competitive ability in territorial interactions [17], resulting in the observed upper limit in song minimum frequency. Our finding of this upper limit on song minimum frequency both within and across dialects points to a need to determine the tradeoffs signalers may face when adjusting their song phenotype to different soundscapes.

In considering the processes that might explain the pattern of variation in song phenotypes, our results suggest that cultural evolution may be the primary mechanism driving spectral variation in song across these soundscapes. In particular, noise level of the site rather than the territory was a better predictor of NWCS song minimum frequency. These results are consistent with cultural evolution because they match a pattern of site-wide homogenization of song that we hypothesize to result from song learning and territory establishment in nearby but different locations. Alternatively, if males adjusted their songs in real time to local ambient noise levels through immediate flexibility alone, we would expect strongest support for models that include territory noise rather than site noise level. We did not find a correlation with territory noise level, even though we sampled across a range of territory noise levels (34.15–70.13 LAeq; 32.9–66 dB(A)) similar to that shown to elicit immediate song adjustment in other species (House Finches 44–65 dB(A) [36]; chiffchaffs 60–66 dB(A) [40]; great tits 60–66 dB(A) [38]; European Blackbirds 40–90 dB(Z) [70]; dB(A) weights for human hearing and dB(Z) is unweighted). Another line of support for cultural evolution as the primary mechanism is our previous finding that white-crowned sparrow songs have increased in song minimum frequency over a thirty year time period in association with increasing ambient noise levels within San Francisco [43, 47]. Our observations suggest that songs in this species appear to be evolving in response to selection pressures from soundscapes. Although our correlative results do not support immediate flexibility as a mechanism, experimental manipulation is needed to determine whether immediate flexibility also influences how songs vary with the soundscape in this species.

Alternatively, the pattern of variation in song minimum frequency could be a by-product of immediate flexibility of song amplitude, known as the Lombard effect [39, 48, 71]. The amplitude and frequency of a sound can be coupled, as phonating birds can increase both amplitude and sound frequency by elevating air pressure at the syrinx (discussed in [72]). In this case, we would expect that territory noise levels would better predict variation in song than site noise levels, as changes in song amplitude would be an immediate response to changes in territory background noise levels. Our finding that site noise level is the best predictor of variation in song minimum frequency suggests that minimum frequency is changing over time independently of song amplitude. It is still possible that some of the variation we find in song minimum frequency is an indirect result of variation in song amplitude. More work is needed in birds to understand the extent to which variation in song minimum frequency is adaptive or an indirect result of an increase in song amplitude [39].

Cultural drift is another possible non-adaptive explanation of variation in song minimum frequency [73, 74]. Variation due to alone drift is unlikely to demonstrate a consistent pattern with respect to noise levels, and the robust pattern of higher minimum frequencies at noisier sites suggests adaptation. However, it is important to note that our results were strongest when examined within three nearby sites producing the same song dialect than across song dialects. This difference in the strength of the relationship to noise suggests that cultural drift [75] and potentially other evolutionary factors play roles in generating additional differences among song dialects that is unrelated to noise levels. This finding further reinforces the need to examine these processes across replicates in order to detect deterministic processes in addition to drift.

Our approach of studying song characteristics in relation to noise levels and masking profiles across multiple sites and dialects is a useful comparative approach that provides robust evidence that soundscapes shape the phenotype of songs and some support that cultural evolution may be the primary driver. In contrast, most work to date suggests that immediate flexibility is the primary mechanism of birds adjusting to variation in their soundscape [14, 38, 40]. However, cultural evolution and immediate flexibility are not mutually exclusive hypotheses, and both may be in effect within species. More work is needed to examine both mechanisms within species to determine their relative roles in shaping the song phenotype in response to selection pressures from auditory masking. Future studies should take into account larger scales of background noise, beyond the immediate territory of the singing bird, in order to examine the potential role of these two mechanisms.

## Supporting Information

S1 File Supporting Data. This is the data set for the manuscript.
(CSV)

S2 File Analyses. This is an R script for all analyses included in the manuscript.
(R) Click here for additional data file.
